# Image‐Guided Magnetic Thermoseed Navigation and Tumor Ablation Using a Magnetic Resonance Imaging System

**DOI:** 10.1002/advs.202105333

**Published:** 2022-02-02

**Authors:** Rebecca R. Baker, Christopher Payne, Yichao Yu, Matin Mohseni, John J. Connell, Fangyu Lin, Ian F. Harrison, Paul Southern, Umesh S. Rudrapatna, Daniel J. Stuckey, Tammy L. Kalber, Bernard Siow, Lewis Thorne, Shonit Punwani, Derek K. Jones, Mark Emberton, Quentin A. Pankhurst, Mark F. Lythgoe

**Affiliations:** ^1^ Centre for Advanced Biomedical Imaging Division of Medicine University College London 72 Huntley Street London WC1E 6DD UK; ^2^ Resonant Circuits Limited 21 Albemarle Street London W1S 4BS UK; ^3^ Cardiff University Brain Research Imaging Centre Maindy Road Cardiff CF24 4HQ UK; ^4^ Victor Horsley Department of Neurosurgery The National Hospital for Neurology and Neurosurgery Queen Square London WC1N 3BG UK; ^5^ Centre for Medical Imaging University College London Charles Bell House, 43‐45 Foley Street London W1W 7TS UK; ^6^ Division of Surgery and Interventional Science University College London Charles Bell House, 43–45 Foley Street London W1W 7JN UK; ^7^ UCL Healthcare Biomagnetics Laboratory University College London 21 Albemarle Street London W1S 4BS UK

**Keywords:** magnetic resonance imaging, magnetic resonance imaging‐compatible, magnetic resonance navigation, minimally invasive, thermoablation

## Abstract

Medical therapies achieve their control at expense to the patient in the form of a range of toxicities, which incur costs and diminish quality of life. Magnetic resonance navigation is an emergent technique that enables image‐guided remote‐control of magnetically labeled therapies and devices in the body, using a magnetic resonance imaging (MRI) system. Minimally INvasive IMage‐guided Ablation (MINIMA), a novel, minimally invasive, MRI‐guided ablation technique, which has the potential to avoid traditional toxicities, is presented. It comprises a thermoseed navigated to a target site using magnetic propulsion gradients generated by an MRI scanner, before inducing localized cell death using an MR‐compatible thermoablative device. The authors demonstrate precise thermoseed imaging and navigation through brain tissue using an MRI system (0.3 mm), and they perform thermoablation in vitro and in vivo within subcutaneous tumors, with the focal ablation volume finely controlled by heating duration. MINIMA is a novel theranostic platform, combining imaging, navigation, and heating to deliver diagnosis and therapy in a single device.

## Graphical abstract

Minimally invasive image‐guided ablation is a novel therapy that uses a magnetic resonance imaging (MRI) scanner to deliver precise focal ablation therapy. A ferromagnetic thermoseed is navigated to the target using the magnetic field gradients generated by the MRI scanner and then heated using an MR‐compatible radiofrequency coil to deliver localized thermoablation.

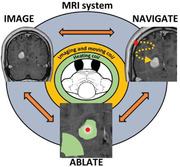

## 1 Introduction

Improving the precision of treatment delivery is arguably the greatest unmet need we have in contemporary medicine.^[^ ^1^, ^2^, ^3^ ^]^ Off‐target effects often result in undesirable toxicities for patients and their associated costs are a burden on healthcare systems. Targeted therapies^[^ ^4^, ^5^, ^6^ ^]^ aim to reduce the harms associated with traditional treatments whilst maintaining control of the disease. For example, targeted drug therapies^[^ ^7^, ^8^ ^]^ and focal ablation techniques^[^ ^9^ ^]^ have been shown to enhance the risk/benefit ratio of cancer therapies, through mitigation of off‐target effects. However, these approaches have tended to be specific to either an organ or disease and, therefore, have had little opportunity to be applied more widely across conditions. In this study, we propose a new technique named Minimally INvasive IMage‐guided Ablation (MINIMA), which uses magnetic resonance navigation (MRN) to steer a therapeutic thermoseed^[^ ^10^ ^]^ through tissue, before delivering localized cell death via thermoablation.

MRN is an emergent technique,^[^ ^11^, ^12^ ^]^ which uses the magnetic field gradients generated by a magnetic resonance imaging (MRI) system to navigate ferromagnetic nanoparticles^[^ ^13^, ^14^ ^]^ or devices^[^ ^15^ ^]^ within the body. MRN is particularly attractive as it enables precise spatial and temporal control over the magnetic field gradients and has the inherent capability of imaging, all of which can aid precise treatment delivery. Preclinical in vivo studies have previously demonstrated the efficacy of MRN to target magnetically labeled cells,^[^ ^13^ ^]^ resulting in increased tumor macrophage infiltration and reduction in tumor burden.^[^ ^14^ ^]^ Alternatively, MRN can be used to steer larger ferromagnetic devices,^[^ ^16^ ^]^ with one proof of concept study navigating an untethered ferromagnetic sphere through the vasculature of a swine.^[^ ^15^ ^]^ Michaud et al. have further developed this concept, with the aim of using microbeads to perform liver embolization.^[^ ^17^ ^]^ These examples are, however, limited to movement within fluid‐filled spaces, such as the cardiovascular system, thus restricting areas within the body that can be reached and the diversity of potential applications.

**Figure 1:**
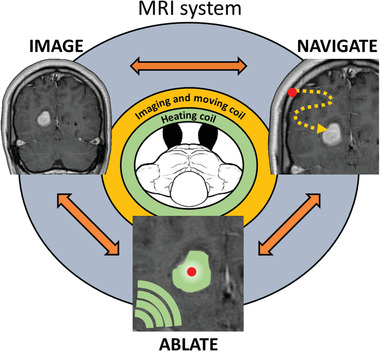
Schematic summarizing the Minimally INvasive IMage‐guided Ablation (MINIMA) concept. IMAGE: MR images will be acquired of the target region from which a path will be planned from insertion site to target, avoiding essential functional tissues. NAVIGATE: A ferromagnetic thermoseed will be inserted at the planned location and navigated along the predetermined path (orange dots) using the magnetic field gradients generated by the MRI scanner. Movement and imaging will be performed in an iterative autonomous sequence, to ensure the thermoseed does not stray from the intended path. ABLATE: Once at the target, ablation periods will be incorporated into the iterative process. This is achieved through the application of an AMF (green arcs), causing the temperature of the thermoseed to increase rapidly and induce localized cell death. Ablation will be performed at multiple locations throughout the target tissue, ensuring the entire tumor volume is ablated. MR image courtesy of Assoc. Prof. Frank Gaillard, Radiopaedia.org, rID: 4090.

Performing MRN directly through tissue will provide more opportunities but is challenging due to the need for larger forces^[^ ^18^, ^19^ ^]^ and the relatively weak magnetic field gradients generated by clinical MRI scanners. This has prompted development of elaborate magnetic technologies such as the gauss gun^[^ ^18^ ^]^ and magnetic hammer actuation,^[^ ^20^ ^]^ but these both lack the control needed for clinical translation. Alternative MRI‐based actuation methods could be used, as these increase the propulsion gradient strength by either introducing ferromagnetic cores into the scanner bore (dipole field navigation^[^ ^21^ ^]^), or by performing the procedure within the fringe field of the magnet (fringe field navigation^[^ ^22^ ^]^). Unfortunately, both methods inhibit the ability to image directly with MRI. Navigation of a ferromagnetic thermoseed through the canine brain has previously been demonstrated, although necessitating purpose‐built electromagnets that can generate gradients of up to 2 T m^−1^.^[^ ^23^, ^24^ ^]^ A technique that could benefit from MRN is magnetic hyperthermia or thermoablation, which aims to induce cell death through the heating of ferromagnetic nanoparticles^[^ ^25^, ^26^ ^]^ or thermoseeds.^[^ ^27^, ^28^, ^29^ ^]^ A major disadvantage of this established technique is the inability to manipulate the particles once inserted into the body, which can result in imprecise treatment delivery and impede its success as a cancer therapy.^[^ ^25^ ^]^ We hypothesize that using a ferromagnetic thermoseed, in tandem with MRI magnetic field gradients specialized for propulsion, we could navigate a thermoseed through tissue to deliver precise thermoablative therapy in situ (**Figure** **1**).

MINIMA has the potential to combine diagnosis and therapy into a single MRI theranostic device, enabling both localization and treatment on the same platform and possibly, in time, at the same sitting. MR images will help determine the least invasive path, along which the thermoseed will be navigated, and using imaging the position of the thermoseed can be constantly assessed, giving real‐time assurance of the thermoseed's location. Once at the target, an alternating magnetic field (AMF) may be applied, causing the thermoseed to heat and deliver localized cell death. The thermoseed may then be navigated through the target tissue, heating at multiple locations until the whole region has been ablated. Having navigated the thermoseed for removal, MRI could then be used to inform on the success of the procedure.

This first evaluation of MINIMA as a minimally invasive focal ablation therapy demonstrates three essential components: imaging, navigation, and therapy. Using an MRI sequence sensitive to ferromagnetic materials,^[^ ^30^, ^31^ ^]^ accurate location of the thermoseed is demonstrated; MRI propulsion gradients are used for precise navigation of the thermoseed in phantoms and brain tissue; and thermoseed heating, using a bespoke hyperthermic device, is shown to induce cell death in cell culture and in vivo within a tumor model. Finally, we present data from a switchable (on/off) MRI scanner, showing that the magnetic field can be removed to ensure safe insertion and extraction of the thermoseed.

## 2 Experimental Section

### 2.1 Agar Preparation

Agar (Sigma Aldrich, USA) was added to distilled water in the desired weight/volume ratio, heated until boiling, poured into the desired container, and left to set at room temperature.

### 2.2 Localization Assessment

A phantom was prepared containing 1 × 0.5 mm diameter thermoseed (AISI 52100, grade 100, Simply Bearings, UK) and 3 × 2 mm diameter plastic spheres. The thermoseed and spheres were positioned at different heights within the Falcon tube by setting a layer of 3% w/v agar solution in a 50 mL Falcon tube, laying a sphere on the surface and pouring unset agar over the top to create the next layer.

Computerized tomography (CT) images were acquired using a nanoScan PET/CT scanner (Mediso, Hungary) with a 50 keV peak X‐ray source and 300 ms exposure time in 720 projections. Images were reconstructed using Nucline software (Mediso, Hungary) to an isotropic voxel size of 251 µm.

MR Images were acquired using a preclinical 9.4 T Bruker Biospec 94/20 MRI scanner (Bruker, Germany). The coordinates of the plastic spheres were determined using a fast spin‐echo (FSE) pulse sequence acquired with the following parameters: repetition time (TR) = 2 s, resolution = 78.1 × 78.1 µm^2^, slice thickness = 1 mm, echo train length (ETL) = 2, receiver bandwidth = 600 kHz, 4 averages. Images were acquired in three orthogonal planes. Coronal and sagittal planes: field of view (FOV) = 80 × 40 mm^2^, matrix size = 1024 × 512, and effective echo time (TE) = 5.12 ms. Axial plane: FOV = 40 × 40 mm^2^, matrix size = 512 × 512, and effective TE = 4.26 ms. The coordinates of the thermoseed were determined using a non‐slice‐selective 1D echo sequence: TR = 1 s, resolution = 78.1 µm, FOV = 80 mm, matrix size = 1024, 8 averages. Radiofrequency (RF) excitation pulse parameters: frequency offset = 3 kHz, bandwidth = 1 kHz. This was repeated twice along each orthogonal axis, once with a positive readout gradient and once with a negative readout gradient.

The center of mass of the plastic spheres in the MR images were determined from a circular region of interest (ROI) drawn over the slice in which the signal void was largest. Each image gave two in‐plane coordinates, which were averaged across the three images to give the final coordinates. The analysis was performed using Analyze tools available in ImageJ.^[^ ^32^ ^]^ For the thermoseed, a cross correlation was performed between the positive and negative gradient projections for each axis. The location of the maximum in the resulting correlation was divided by two to give the coordinates of the thermoseed with respect to the isocenter of the magnet. This analysis was performed using a custom script in MATLAB (MathWorks, USA). In the CT images, the in‐plane coordinates for each sphere were determined from a circular ROI drawn over the slice in which the sphere appeared largest. Two in‐plane coordinates were determined for each orthogonal plane and averaged across the three planes to give the final coordinates. This analysis was also performed in ImageJ.

### 2.3 Navigation through Agar

A 0.5 or 1 mm diameter thermoseed was placed in a 22 × 25 × 25 mm^3^ container filled with 0.125% w/v agar. The sample was positioned in the scanner bore and its temperature was maintained at 22 °C using an air‐pump feedback control system.

MR images were acquired using a preclinical 9.4 T Varian Inova MRI scanner (Varian, USA) with a 39 mm Rapid RF volume coil (Rapid Biomedical, Germany). The position of the thermoseed was monitored using a gradient‐echo pulse sequence: TR = 15.428 ms, TE = 0.69 ms, FOV = 25.6 × 25.6 mm^2^, matrix size = 64 × 64, slice thickness = 2 mm.

To perform the movement experiments, the gradient‐echo sequence was edited to include propulsion gradients prior to image acquisition. Controllable parameters were gradient strength, direction, duty cycle (on/off time), and number of loops. This sequence was used to perform all preclinical movement experiments performed on the Varian Inova MRI scanner. To deliver the magnetic field gradients, a 60 mm diameter bore imaging gradient coil insert (Varian, USA) was used. Due to cooling restrictions of the gradient coils, a duty cycle of 2/7 ms on/off (22%) was used with the number of loops kept at 500, delivering an effective on time of 1 s. The gradient strength was varied between 1 and 500 mT m^−1^. The distance moved between images was measured manually using ImageJ.

### 2.4 Assessing Directional Control and the Effect of Duty Cycle and Thermoseed Material

A 2 mm diameter thermoseed was placed in a 22 × 25 × 25 mm^3^ container filled with golden syrup (an inverted sugar syrup made by Tate & Lyle, UK). A layer of water was added to the surface of the golden syrup to improve signal‐to‐noise in the MR images. Once positioned in the scanner bore, the sample temperature was maintained at 22 °C. Propulsion parameters used for each experiment are detailed in **Table** **1**.

**Table 1:** Propulsion parameters used for navigation characterization experiments

Experiment	Gradient strength [mT m^−1^]	Duty cycle [ms on/off]	Number of loops	Total gradient on time [s]
Directional control (Figure 3b)	500	2/7	500–2000	1–4
Gradient strength in sugar syrup (Figure S1b, Supporting Information)	50–1000	2/7	2000	4
Number of loops (Figure S1c, Supporting Information)	500	2/7	250–2000	0.5–4
Duty cycle (Figure S1d, Supporting Information)	400	2/7	2000	4
		2/2	4500	9
		2/0	9000	18
Material (Figure S1e, Supporting Information)	50–400	1/0	10	10
0.25 mm thermoseed (Figure S2, Supporting Information)	979	100/900	15	1.5
			16	1.6
			20	2
			40	4

An established method^[^ ^30^, ^31^ ^]^ was adapted to measure the displacement of the thermoseed using a 2D non‐slice‐selective spin‐echo pulse sequence: TR = 0.5 s, TE = 13.8 ms, ETL = 4, FOV = 50 × 35 mm^2^, matrix size = 128 × 128, slice thickness = 30 mm, 1 average, RF excitation bandwidth = 2 kHz, RF excitation frequency offset = 30 kHz. 1D projections along the readout and phase encoding directions were obtained by summating along the image rows and columns. The mode pixel value was then subtracted from each pixel in the 1D projection. The relative movement along each axis was calculated by cross correlating the 1D projections acquired prior to and following each movement.

### 2.5 Assessing Effect of Gradient Strength and Number of Loops

A thermoseed (0.5–3 mm diameter) was placed in a 25 × 50 mm^2^ rectangular box filled with golden syrup to a depth of 5 mm.

Experiments were performed using a shielded BFG 102/60‐S13 gradient set, capable of generating magnetic field gradients of up to 979 mT m^−1^, inserted into a 9.4 T Bruker Biospec MRI system. The sphere was moved horizontally through the viscous medium, with six repetitions performed for each parameter value. The propulsion sequence consisted of a single gradient pulse followed by an interval, looped to achieve a total gradient on time of 4 s (Figure S1a, Supporting Information). Propulsion parameters used for each experiment are detailed in Table 1.

Displacement of the thermoseed was measured using 1D projection images as described for the localization assessment. 1D imaging parameters: TR = 2 s, resolution = 117.2 µm, FOV = 60 mm, matrix size = 512, 1/8 averages. RF excitation pulse parameters: frequency offset = 5–40 kHz, bandwidth = 6–36 kHz. Images acquired prior to and following each movement were cross correlated to determine the relative displacement.

### 2.6 Navigation of a 0.25 mm Thermoseed

A 0.25 mm thermoseed was added to a 22 × 25 × 25 mm^3^ container filled with 0.1% w/v agar. Experiments were performed using 9.4 T Bruker Biospec 94/20 MRI scanner fitted with a BFG 102/60‐S13 gradient insert. The thermoseed was navigated in a zig–zag pattern in the horizontal plane. Propulsion parameters are detailed in Table 1. 2D images were acquired at regular intervals to track the moment of the thermoseed, using a fast low angle short sequence. Imaging parameters: TR = 0.1 s, TE = 2.5 ms, resolution = 0.47 × 0.23 µm^2^, slice thickness = 0.5 mm, FOV = 60 × 30 mm^2^, matrix size = 128 × 128, receiver bandwidth = 50 kHz, 1 average.

### 2.7 Navigation along a Three‐Dimensional Path

A 1 mm thermoseed was added to a 74 × 21 × 21 mm^3^ container filled with 0.25% w/v agar. Experiments were performed using 9.4 T Bruker Biospec 94/20 MRI scanner. The thermoseed was navigated along a 3D path using the propulsion parameters detailed in Table 1, and the displacement of the thermoseed was measured by cross‐correlating 1D projections acquired before and after each movement. Imaging parameters: TR = 2 s, resolution = 312.5 µm, FOV = 80 mm, matrix size = 256, 1 average. RF excitation pulse parameters: frequency offset = 10 kHz, bandwidth = 4 kHz.

### 2.8 Navigation through Ex Vivo Brain Tissue

Pig brains were obtained locally at Meat16 (UK). The maximum postmortem time before experiments was 5 days. Samples were refrigerated and kept in phosphate‐buffered saline (PBS) prior to experiments. To prepare the samples, the front of the hemisphere was cut to fit within the dimensions of the container (22 × 25 × 25 mm^3^), placed inside and filled with PBS. Samples were incubated at 37 °C for 1 h prior to experiments and positioned in a 39 mm Rapid RF volume coil, with temperature monitored and maintained at 37 °C throughout the experiment. 2 mm diameter thermoseeds and the following propulsion parameters were used throughout: duty cycle = 20/70 ms on/off, number of loops = 500–5000, gradient strength = 300–500 mT m^−1^.

Prior to insertion of the thermoseed, an image was acquired using an FSE pulse sequence: TR = 1 s, effective TE = 20 ms, slice thickness = 1 mm, matrix size = 256 × 256, number of slices = 20. To measure the displacement of the thermoseed, the same 2D non‐slice‐selective spin‐echo sequence and parameters were used as for the initial golden syrup experiments (Figure 3). 1D projections were also obtained in the same way. The relative position of the thermoseed was taken as the first (or last, depending on the movement direction) pixel ≥ half the maximum pixel intensity in each projection. These positions were then placed on a 22 × 25 mm^2^ grid and overlaid onto the pre‐acquired MR image. The edges of the grid were manually adjusted to fit the edges of the box on the MR image.

### 2.9 Navigation Experiments in a Human Magnetic Resonance Imaging Scanner

Experiments were performed using a 3 T Siemens scanner (Germany) fitted with a Connectom gradient set at the CUBRIC Centre in Cardiff. The specifications for the Connectom gradient coil were maximum gradient strength = 300 mT m^−1^, slew rate = 200 T m^−1^ s^−1^, inner bore diameter = 56 cm, and imaging volume = 20 cm diameter spherical volume with 6% gradient linearity. A 3 mm diameter thermoseed was placed in a 200 × 120 × 70 mm^3^ container filled with 0.125% w/v agar.

The position of the thermoseed was monitored using a gradient‐echo pulse sequence: TR = 8.6 s, TE = 4 ms, ETL = 1, FOV = 250 × 250 mm^2^, matrix size = 512 × 512, resolution = 0.49 × 0.49 mm^2^, slice thickness = 7 mm, 2 averages. Propulsion parameters: duty cycle = 15/85 ms on/off (15%), number of loops = 2, gradient strength = 100–280 mT m^−1^. Distance moved between images was measured manually in MATLAB.

### 2.10 Navigation through Ex Vivo Brain Tissue Using Clinically Relevant Propulsion Parameters

A section of ex vivo pig brain tissue was cut to the size of a 35 × 60 × 12 mm^3^ container, placed inside and filled with PBS. Samples were incubated at 37 °C for 1 h prior to experiments and positioned in a 39 mm Rapid RF volume coil, with temperature monitored and maintained at 37 °C. 3 mm diameter thermoseeds were used throughout.

To perform the movement experiments a custom‐built propulsion gradient set was developed (Tesla Engineering, UK), capable of generating up to 500 mT m^−1^ continuously (100% duty cycle), as well as operating as a standard imaging gradient coil (Figure S6, Supporting Information). Two sets of propulsion parameters were used for these experiments. Set 1: duty cycle = 15/85 ms on/off (15%), gradient strength = 400 mT m^−1^. Set 2: duty cycle = 5/0 s on/off (100%), gradient strength = 300 mT m^−1^.

Prior to insertion of the thermoseed, a pre‐image was acquired using an FSE pulse sequence: TR = 1 s, effective TE = 9 ms, ETL = 4, FOV = 40 × 40 mm^2^, matrix size = 256 × 256, slice thickness = 1 mm, 1 average. To measure the displacement of the thermoseed, the same imaging sequence and processing was used as for the previous ex vivo brain tissue experiments.

### 2.11 Heating Experiments

For all heating experiments, a custom‐built MR compatible magnetic alternating current hyperthermia (MACH) system (Resonant Circuit Ltd., UK) was used to generate an AMF of up to 8 kA m^−1^ at 700 kHz.

### 2.12 Heating in Air

A thermoseed (1–3 mm diameter) was positioned on the tip of a fiber‐optic temperature probe (FOTEMP1‐OEM, Weidmann, Germany), inside the MACH system. The AMF was then applied for 1 min. The temperature of the thermoseed was recorded throughout the heating and until the thermoseed had returned to room temperature. This was repeated three times for each thermoseed size.

### 2.13 Thermoablation in Three‐Dimensional Cell Culture

The human colorectal adenocarcinoma cell line SW1222, previously stably transduced with lentiviral vector to express firefly luciferase enzyme (Fluc), were grown in T175 flasks (Fisher Scientific, UK) in Dulbecco's Modified Eagle Medium (DMEM) supplemented with 10% fetal bovine serum (FBS, Invitrogen, UK), in a humidified incubator at 37 °C with 95% air and 5% CO_2_. Cells were grown to 80% confluence before being trypsinized, counted, and centrifuged for pelleting. A 0.65% w/v, 40 °C agar solution was added to resuspend the cells to a concentration of 40 000 000 cells per mL. Circular plastic cups (21.2 mm in diameter) were cleaned and dried, and 0.9 mL of the cell suspension was added to each cup. After the gel had set, 0.5 mL of medium (DMEM with no phenol red, 10% FBS) was added to each cup and the 3D cell cultures were kept in the incubator until use.

After a 2 mm diameter thermoseed was placed in the middle of a 3D cell culture, the AMF was applied for 0, 1, or 5 min. After heating, 2 mL of 1.5% 2,3,5‐Triphenyltetrazolium chloride (TTC) solution (w/v, Sigma Aldrich, USA) was applied to the cells and incubated at 37 °C for 30 min. The TTC was then removed, and photos of the cell cultures were taken. The cross‐sectional area of cell death was calculated in ImageJ by thresholding the image.

To measure the temperature at 0, 1, 2, and 3 mm away from the thermoseed's surface, a fiber optic temperature probe (Luxtron Co., USA) was placed in a 0.5% w/v agar phantom. Measurements were taken every second for 10 min, plus 1 min after the AMF was turned off. At least two replicates were acquired at each distance.

For experiments performed in the MRI scanner, a 15 mL Falcon tube was prepared by filling with 1% w/v agar and embedding a 2 mm diameter thermoseed in the center. A fiber optic temperature probe was placed on the surface of the thermoseed.

### 2.14 Thermoablation in the Rat Brain

All animal work was approved by the University College London internal Animal Welfare and Ethical Review Body and licensed under the UK's Animals (Scientific Procedures) Act of 1986 (PPL 70/8421, PPL 70/7496). Male Wistar rats (8 ± 1 weeks‐of‐age) were bred by Charles River (UK) and delivered to UCL's Centre for Advanced Biomedical Imaging 1 week prior to experiments. Rats (300 ± 25 g) were anaesthetized with 3.5% isoflurane vaporized in oxygen at a flow rate of 1 L min^−1^. The head was shaved, and the animal was positioned in a stereotaxic frame in the horizontal skull position, where 2.5% isoflurane (delivered in 1 L min^−1^ oxygen) was delivered via a nose cone. A midline incision was made in the scalp and a cranial window made by removing a ≈6 mm disc of skull above the left striatum (3 mm lateral and 0.2 mm anterior to bregma). A 27‐gauge needle was inserted into the striatum (6 mm ventral to the brain surface) and removed. This process was subsequently repeated at the same position with a 21‐ and 18‐gauge needle. A 2 mm diameter thermoseed was positioned on the surface of the brain, above the needle tract. The thermoseed was inserted into the striatum using a blunt 18‐gauge needle, prior to the needle being retracted and the skin sutured closed to cover the skull.

The rat was transferred to the MRI‐compatible MACH system, with the head positioning in the center of the coil, and anesthetic maintained via a nose cone (2.5% isoflurane delivered in 1 L min^−1^ oxygen). The AMF was applied for 1 min (8 kA m^−1^, 700 kHz).

Following heating, the animal was sacrificed with an overdose of pentobarbital administered via intraperitoneal injection. The animal was decapitated, sutures removed, and the thermoseed extracted through the original needle tract using a permanent magnet. The brain was removed from the skull and sectioned into 1 mm thick slices using a McIlwain tissue chopper (Mickle Laboratory Engineering Co. Ltd., UK). Each slice was placed into freshly prepared 1% TTC solution and incubated at 37 °C for 10 min. The slices were removed from solution, excess liquid removed, and placed on a petri dish. Samples were photographed immediately. ROIs were manually drawn around the area of cell death in ImageJ.

### 2.15 Thermoablation in a Subcutaneous Tumor Model

The SW1222*^Fluc^* cells were trypsinized, counted, centrifuged for pelleting, and resuspended in PBS for in vivo cell injection.

Female CD‐1 *nu/nu* mice (6–8 weeks old, 25–30 g) were subcutaneously injected on the right flank with 2.5 × 10^6^ SW1222 cells in 100 µL of PBS. Tumor growth was monitored every 3–4 days using bioluminescence imaging (BLI) and caliper measurements. Measurements were taken in three orthogonal directions and tumor volumes were estimated using the formula *V* = *l* × *w* × *h* × *π*/6.

The mice were anaesthetized for all procedures with 4% isoflurane in 1 L min^−1^ oxygen and maintained at 2% isoflurane. A 19‐gauge needle was inserted into the center of the tumor and removed to create a tract. A 1.5 or 2 mm diameter thermoseed was inserted through the tract, into the tumor, using a blunt 19‐gauge needle. The animal was transferred to the MRI‐compatible MACH coil and the AMF was applied for 1 × 1, 3 × 1, or 5 × 1 min periods separated by 1 min intervals (8 kA m^−1^, 710 kHz). Following ablation, the thermoseed was extracted through the original tract using a permanent magnet.

### 2.16 Bioluminescence Imaging

Following insertion of the thermoseed, mice (5 × 1 min heating) were administered intraperitoneally with 120 mg kg^−1^ of D‐luciferin in 200 µL of PBS and placed in the imaging chamber. Mice were positioned on their side with the tumor facing up toward the camera. Images were acquired immediately prior to and post heating, with the thermoseed in place. In addition, BLI was performed up to 7 days prior to and 10 days post treatment (Figure 6a). Animals were administered with D‐luciferin as described above, and images were acquired from 8 to 38 min following injection, at 1 min intervals. The maximum luminescence reading for each stability curve was used for each time point.

### 2.17 Histology

Mice were sacrificed 15 min (1 min heating) or 24 h (3 × 1 min heating) post heating by cervical dislocation and the tumors removed for histology. Tumors were fixed in 4% formalin, dehydrated in 70% ethanol, and embedded in paraffin wax blocks. Tumors were sectioned at 3 µm and stained with hematoxylin for 1 min and eosin for 30 s using an automated slide stainer (Sakura, Netherlands). Sections were imaged using a NanoZoomer Digital Scanner and analysis software NDP.view2 (Hamamatsu, Japan). Images were processed in ImageJ using the NDPITools plugin.^[^ ^33^ ^]^ The area of necrosis was segmented manually.

### 2.18 Ramping Magnet Experiments

Experiments were performed on an MRS Magnetics 3 T 170 mm clear bore magnet (MR solutions, UK). 0.3% w/v agar solution was prepared by dissolving 0.3 g agar in 100 mL of deionized water and heated until boiling. 0.5 g of ivory black pigment (L. Cornelissen & Son, UK) was added to the solution and thoroughly mixed. Approximately 6 mL of the solution was added to 35 × 10 mm petri dishes. The samples were left to set at room temperature, allowing the pigment to settle at the bottom of the dish.

Immediately prior to each experiment, a 3 mm diameter thermoseed was inserted into the center of the sample, resting on the bottom of the dish. The sample was then positioned within the FOV of the magnet, whilst at 0 T. The magnetic field was ramped up to 3 T, held for 2 min and ramped back down to 0 T. To ensure movement could be detected with the designed phantom, control samples were positioned 13 cm from the isocenter and the ramping protocol was repeated. Three replicates were performed for each set up. Photographs were taken prior to and immediately following each experiment.

### 2.19 Statistical Analysis

All data were expressed as the mean ± 1 standard deviation. For in vivo heating data, repeated measures two‐way ANOVA testing, followed by a Bonferroni post‐hoc test was carried out across groups. Linear regression analysis was used to determine the effect of exploratory variables on the distance moved by the thermoseed in all navigation experiments. In all cases, significance was defined as *p* ≤ 0.05. Statistical analysis was performed in GraphPad Prism v.8.3 or IBM SPSS Statistics 27.

## 3 Results

### 3.1 Locating the Thermoseed

**Figure 2:**
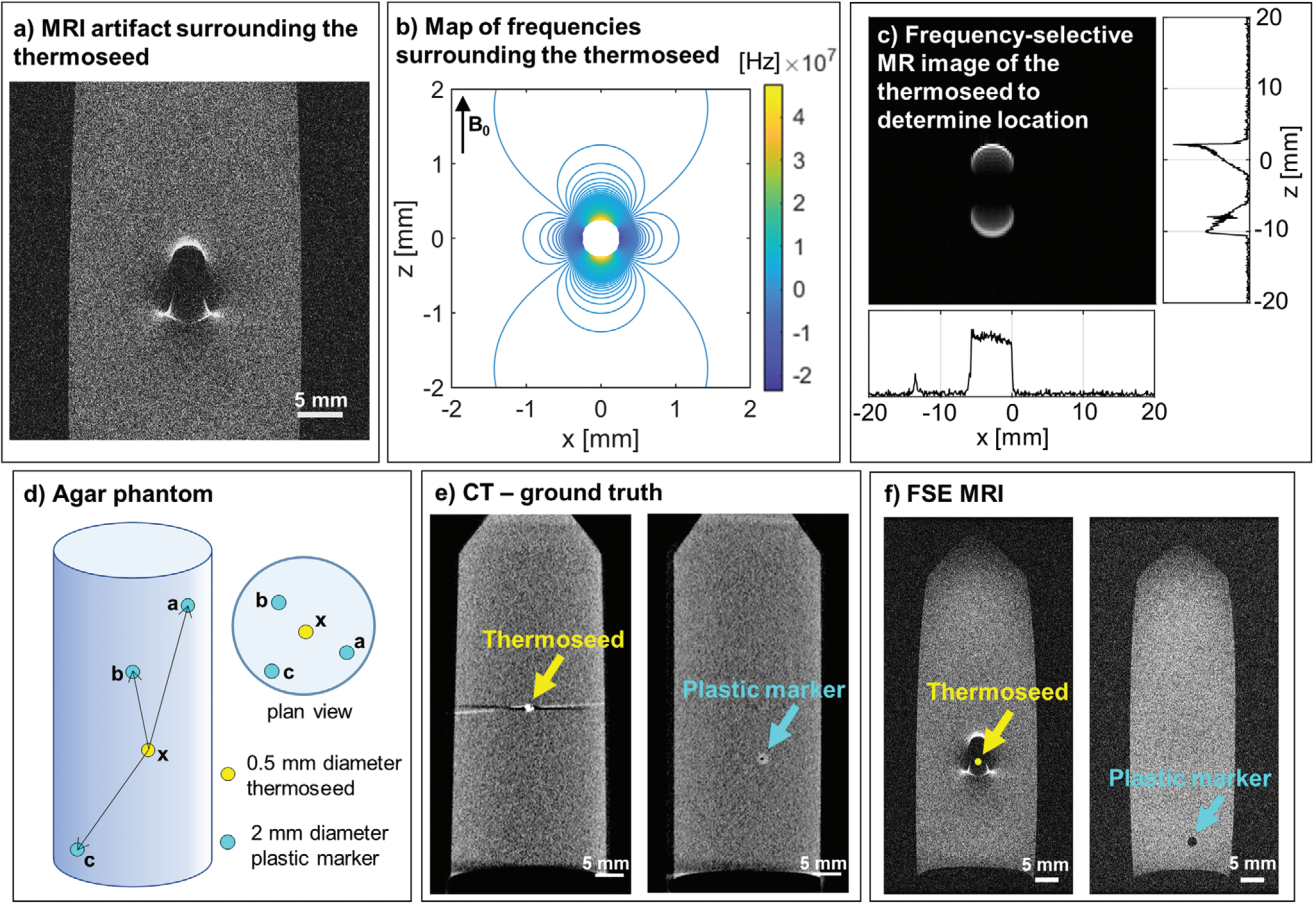
Locating the thermoseed. a) Distortion MRI artifact caused by the ferromagnetic properties of the thermoseed. Image of a 0.5 mm diameter chrome steel sphere acquired with a fast spin‐echo pulse sequence. b) Simulated map of resonance frequencies surrounding a 0.5 mm diameter thermoseed. c) Discrete bands can be selectively excited using a frequency‐selective MRI pulse sequence.^[^ ^30^, ^31^ ^]^ The signal can be projected onto a 2D plane or 1D axis, from which the thermoseed's location can be determined. d) An agar phantom consisting of three plastic fiducial markers and one thermoseed was used to assess the accuracy of the frequency‐selective MRI method. e) Ground truth distances between each thermoseed‐marker pair were calculated from CT images. f) Marker coordinates were determined from fast spin‐echo MR images. Thermoseed coordinates were calculated using the frequency‐selective MRI method, and thermoseed‐marker distances were compared with CT values to assess method accuracy.

MINIMA requires the ability to locate the thermoseed with a high degree of accuracy. However, due to image distortion artifacts caused by the ferromagnetic properties of the thermoseed (**Figure** **2**a), traditional MRI sequences cannot be used. To overcome this, a frequency‐selective MRI method was adopted,^[^ ^30^, ^31^ ^]^ which takes advantage of the magnetic field pattern around the thermoseed (Figure 2b). This pattern contains discrete frequency bands that can be selectively excited during MRI. The resulting signal can be displayed as a 2D or 1D projection and used to identify the location of the thermoseed (Figure 2c).^[^ ^30^, ^31^ ^]^

To assess the accuracy of thermoseed localization, we compared the MRI method with CT using fiducial markers embedded in a phantom. The agar phantom comprised three plastic markers (MRI and CT visible) set at varying distances from the thermoseed (Figure 2d–f). The thermoseed was localized using the frequency‐selective MRI method and the plastic markers using an MR spin‐echo image (Figure 2f). From this, the distance between each thermoseed‐marker pair (ax, bx, cx) was calculated and compared with ground truth values taken from CT images (Figure 2e). The mean thermoseed localization error was no more than 0.27 ± 0.08 mm (*n* = 5, **Table** **2**), demonstrating the ability to image with submillimeter accuracy, an essential requirement for in vivo navigation.

**Table 2:** Accuracy of locating the thermoseed. Difference between thermoseed‐marker pair distances measured using MRI and CT. Data shown as mean ± S.D.; *n* = 5

Thermoseed‐marker pair	Difference [mm]
ax	0.27 ± 0.08
bx	0.02 ± 0.12
cx	0.17 ± 0.13

### 3.2 Navigating the Thermoseed

**Figure 3:**
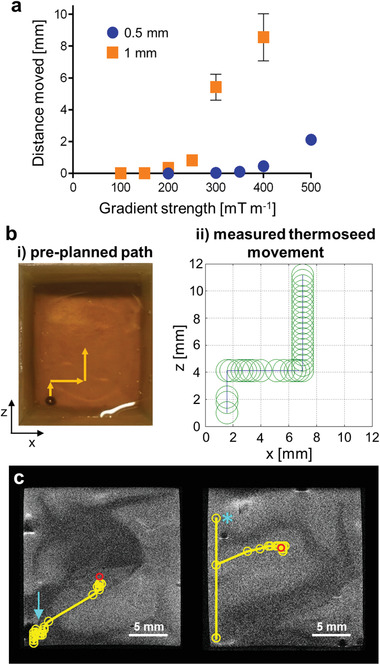
Initial navigation experiments in agar, sugar syrup, and ex vivo porcine brain tissue. a) Distance moved versus magnetic field gradient strength for 0.5 and 1 mm diameter thermoseeds in 0.125% agar (duty cycle = 2/7 ms on/off; number of loops = 500). An increase in distance moved was observed with increasing magnetic field gradient strength, as well as thermoseed size. Data shown as mean ± S.D., *n* ≥ 3. b) To assess directional control, a 2 mm diameter thermoseed was navigated along a preplanned path (i, +3 mm *z*, +7 mm *x*, +7 mm *z*), through a viscous medium (sugar syrup). The measured displacements (ii) followed the intended path without deviation. Each circle represents an individual movement. (Gradient strength = 500 mT m^−1^; duty cycle = 2/7 ms on/off; number of loops = 500–2000). c) Navigation demonstrated through two ex vivo brain tissue samples using a 2 mm thermoseed, overlaid onto pre‐acquired T_2_‐weighted MR images. Each yellow circle represents an individual movement. In the first sample an intervening tissue structure was circumnavigated (arrow) before continuing to the final location (circle) as intended. In the second sample the initial movement overshot (star), therefore, the thermoseed was traced back along the path and directed toward the final location (circle). (Gradient strength = 300–500 mT m^−1^; duty cycle = 20/70 ms on/off; number of loops = 500–5000).

To deliver therapy to precise locations within the body, we propose navigating the ferromagnetic thermoseed through tissue, using the magnetic field gradients generated by an MRI scanner. The parameters through which the magnetic field can be controlled are the gradient strength, the duty cycle (ratio of on/off time), number of loops (repetitions of the on/off cycle), and the direction (Figure S1a, Supporting Information). Our first aim was to establish whether distance moved was dependent on gradient strength and thermoseed diameter. The duty cycle was kept constant at 2/7 ms on/off (22%) throughout the experiment, with 500 loops. By navigating thermoseeds through an agar phantom, we observed that increasing the gradient strength increased the distance moved. For example, below 200 mT m^−1^ no movement was observed, whereas clear movement (8.6 ± 1.5 mm) was detected at 400 mT m^−1^ (**Figure** **3**a). Thermoseed movement was achieved at gradients below 200 mT m^−1^ when using larger thermoseeds (Figure S1b, Supporting Information), and movement with thermoseeds as small as 0.25 mm was also possible (Figure S2, Supporting Information). In addition to thermoseed size, an increased number of periods, higher duty cycle, and alternative thermoseed material also produced an increase in movement (Figure S1, Supporting Information).

We investigated directional control by navigating a thermoseed along a predetermined path through a homogenous viscous medium (sugar syrup). The planned path followed a stepwise pattern in the *xz* plane (Figure 3b[i]). The thermoseed's position was maintained along this preplanned path throughout the experiment, demonstrating precise navigation in both magnitude and direction (Figure 3b[ii]). Navigation was also performed along a 3D path in agar (Figure S3, Supporting Information).

Next, we investigated the ability to move the thermoseed through ex vivo porcine brain tissue, with the aim of navigating a 2 mm thermoseed from the corner to the center of the sample (Figure 3c, red circle). The movement of the thermoseed was overlaid onto anatomical T_2_‐weighted images showing grey and white matter boundaries. In the first sample, movement was interrupted by an intervening structure (Figure 3c, blue arrow). In response, the direction of movement was altered by 90°, allowing the tissue to be circumnavigated, before continuing along the planned trajectory. In the next sample, the initial applied force caused the thermoseed to overshoot the preplanned path (Figure 3c, blue star). Based on the imaging data, the path was retraced before moving to the sample center. The unplanned deviations emphasize the importance of incorporating imaging into the procedure. By adopting an iterative process of moving and imaging, the applied forced can be adjusted based on continuous assessment of the movement, thus maintaining a high level of control throughout.

### 3.3 Clinical Feasibility

**Figure 4:**
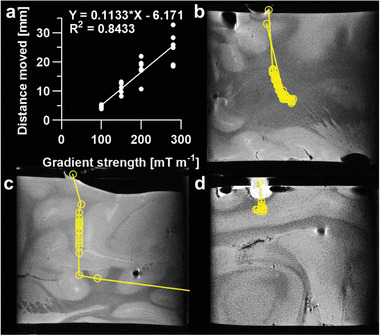
Navigation using clinically relevant magnetic field gradient strengths. a) Distance moved versus magnetic field gradient strength for a 3 mm thermoseed through 0.125% agar, using a Connectom system designed for human imaging. Distance moved increased with increasing gradient strength. Data analyzed using simple linear regression, *n* = 6 measurements for each gradient strength, *p* < 0.0001. b,c) A 3 mm thermoseed was navigated through ex vivo porcine brain tissue with a clinical gradient strength of 300 mT m^−1^ (maximum limit of the Connectom system) and duty cycle of 100%. The thermoseed was navigated 45 and 53 mm in samples B and C, respectively. d) A 3 mm thermoseed was navigated 8 mm through ex vivo brain tissue, with a gradient strength of 400 mT m^−1^ and a clinical duty cycle of 15% (maximum limit of the Connectom system). Movement was most efficient when using a clinically relevant gradient strength of 300 mT m^−1^ applied continuously. Each circle represents an individual movement.

To demonstrate feasibility and control of movement using an MRI scanner designed for human imaging, a 3 mm thermoseed was navigated using a Connectom system (Siemens Healthineers) with a maximum gradient strength of 300 mT m^−1^.^[^ ^34^, ^35^ ^]^ The maximum duty cycle was 15/85 ms on/off (15%) and was kept constant throughout. As with the data acquired using a preclinical system (Figure 3a), increased gradient strength caused an increase in movement (**Figure** **4**a); 280 mT m^−1^ resulted in a movement of 24.9 ± 5.4 mm (*n* = 6), compared to 4.6 ± 0.7 mm (*n* = 6) at 100 mT m^−1^. These data clearly demonstrate the potential to navigate and control a thermoseed using an MRI system in clinical practice.

For clinical translation, existing MRI scanners with standard imaging gradients (40–80 mT m^−1^) would require a bespoke propulsion gradient coil, the two main specifications for which are the gradient strength and duty cycle. With this in mind, two further experiments were performed in porcine brain tissue on a preclinical MRI system, using either gradient strength or duty cycle values achievable on the Connectom system. The thermoseed was navigated a total of 45 mm (Figure 4b) and 53 mm (Figure 4c) in 66 and 8 min, respectively, using 300 mT m^−1^ (duty cycle 100%). The difference in duration was largely due to the tissue through which the thermoseed was travelling, white matter in sample B (Figure 4b) and grey matter in sample C (Figure 4c), which have different elastic moduli.^[^ ^36^ ^]^ Additionally, in sample B, the time spent returning to the start point accounted for just 11% of the total duration, demonstrating the ease with which the thermoseed can be returned to the injection site for retrieval. Furthermore, using a duty cycle of just 15% (400 mT m^−1^) we still achieved 8 mm of movement through brain tissue (45 min, Figure 4d). These data indicate that with continuous application of the gradient, a 3 mm thermoseed can be moved through brain tissue on systems designed for human imaging.

Another key challenge for clinical translation is the safe insertion and removal of the thermoseed, due to the fringe field gradients at the entrance of the bore. One solution is to switch off the magnetic field until the patient is positioned within the scanner, which we investigated using a preclinical, rampable MRI system. An agar phantom containing a 3 mm thermoseed was used to ensure that no significant forces would be exerted on the thermoseed during the ramping process. With the phantom positioned within the FOV of the scanner, the magnet was ramped from 0 to 3 T within 7.5 min and back down again. No movement was observed in any of the three replicates (Figure S4, Supporting Information). In contrast, control samples positioned outside the FOV demonstrated that movement could readily be detected with the phantom used. This result supports the use of a rampable magnet for the safe insertion and removal of the thermoseed during the timescale of the procedure.

### 3.4 Heating the Thermoseed to Induce Cell Death

**Figure 5:**
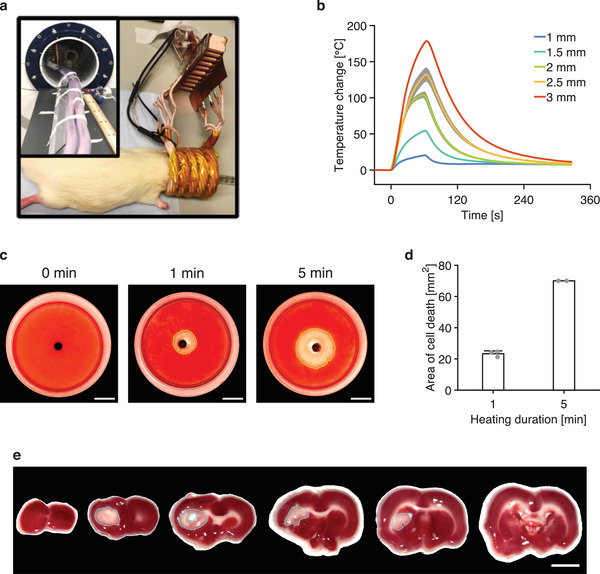
Heating the thermoseed in vitro and in vivo. a) Position of thermoablative device around a rodent's head. Insert: Thermoablative device placed inside the bore of the MRI scanner. b) Change in temperature on the surface of thermoseeds of different sizes as they were heated by the thermoablative device in air. Data shown as mean ± S.D., *n* = 3. c) Photos of TTC stained 3D cell cultures following 0, 1, and 5 min of heating with a 2 mm thermoseed show a clear perimeter of cell death (red = alive cells, clear = dead cells). Scale bar = 5 mm. d) Cross‐sectional area of cell death for different heating durations. A longer duration of heating causes a larger area of cell death. *n* ≥ 2 measurements for each heating duration. e) Rat brain slices stained with TTC solution following 1 min of heating with a 2 mm thermoseed show a well‐defined area of cell death (blue ROI). Scale bar = 5 mm.

Once the thermoseed has been navigated to the tumor, thermoablation therapy will be delivered by applying an external AMF using a bespoke, MR‐compatible hyperthermic device (**Figure** **5**a). We first measured the surface temperature of a series of thermoseeds (1–3 mm in diameter) as they were heated by the hyperthermic device (700 kHz, 8 kA m^−1^) in air. Temperature changes greater than 50 °C were readily achieved within 30 s when the thermoseed was 2 mm in size or larger (Figure 5b). Further investigation of 2 mm thermoseeds showed that the heating effect diminished with distance from the thermoseed surface (Figure S5a, Supporting Information). For instance, at 1 mm from the thermoseed, it would take 45 s to achieve an 18 °C increase, which would heat tissue from 37 to 55 °C, the critical temperature for rapid ablation,^[^ ^37^ ^]^ whereas at 2 and 3 mm from the surface, a heating duration of 186 and 377 s, respectively, would be required to achieve the same (Figure S5a, Supporting Information). When we performed the same experiments in an MRI scanner, a temperature increase of 27 °C was recorded after 30 s of heating (Figure S5b, Supporting Information), which is similar to the thermoseed surface temperature obtained following 30 s of heating on the bench (27 °C, Figure S5a, Supporting Information).

Next, to assess the effect of hyperthermia on cancer cells, we placed a 2 mm thermoseed in the center of a 3D cell culture and heated it for 1 or 5 min to assess cell death. After 1 min of heating, a clear perimeter of cell death was observed, while no visible cell death occurred in the control sample (Figure 5c). The cross‐sectional area of cell death was 23.3 ± 1.8 mm^2^ (equivalent to a spherical volume of 84.8 ± 9.9 mm^3^ with a diameter of 5.5 ± 0.2 mm) and 70.0 ± 0.0 mm^2^ (equivalent to a spherical volume of 440.9 ± 0.4 mm^3^ with a diameter of 9.4 ± 0.0 mm) after 1 and 5 min of heating, respectively (Figure 5d). These results show that cell death is not only possible, but the extent of cell death can be tightly controlled.

When performing ablation in vivo, additional biological processes, such as blood flow, may dissipate heat and inhibit the ability to induce cell death. To investigate this, we inserted a 2 mm thermoseed into the left striatum of the rat brain and heated for 1 min (700 kHz, 8 kA m^−1^). We observed marked cell death (Figure 5e) within the grey matter of the striatum (area of cell death = 12.3 mm^2^), indicating the potential to perform tissue ablation in vivo. The area decreased with each neighboring slice, forming a spheroid of cell death around the thermoseed.

**Figure 6:**
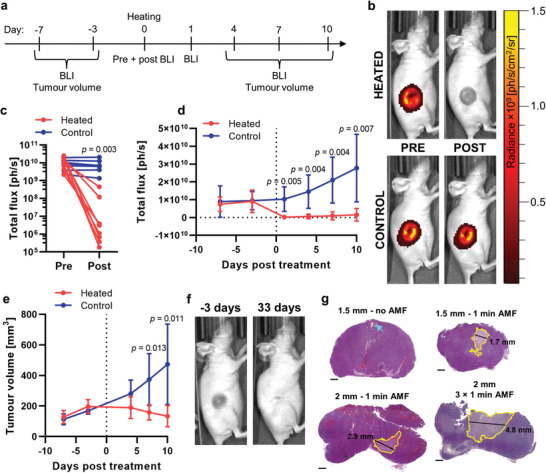
Thermal ablation in a mouse subcutaneous tumor model. a) Experimental timeline of BLI, tumor volume measurements, and heating. b) Bioluminescence images of a heated SW1222*^Fluc^* tumor acquired immediately prior to and following heating and a nonheated control tumor, with a thermoseed in place. c) BLI signal integrated over the whole tumor decreased in the treated animals and remained constant for the controls. *n* = 8 and 7 for the heated and control groups, respectively. d) BLI signal measured longitudinally for treated and control animals, acquired with the thermoseed removed. e) Tumor volume measurements recorded over the course of the experiment. f) Photographs of a treated tumor 3 days prior to and 33 days post treatment show complete ablation of the tumor bulk. g) Hematoxylin and eosin staining of sections of subcutaneous tumors ablated with a 1.5 or 2 mm thermoseed for 1 min or 3 × 1 min show clear regions of necrosis (yellow ROI). The control sample has no distinct area of necrosis but does show the needle insertion tract (blue arrow). Scale bar = 1 mm. In panels (d,e): data shown as mean ± S.D., *n* = 6 and 7 for heated and control groups, respectively. In panels (c–e): *p*‐values are calculated using two‐way repeated measures ANOVA with Bonferroni correction.

Last, we assessed the efficacy of this thermoablation technique as a cancer therapy. A murine subcutaneous tumor model was established with the SW1222*^Fluc^* cell line that expresses firefly luciferase (Fluc). Tumor development was monitored with BLI and volume measurement for a period of 17 days, starting from 7 days before the treatment (**Figure** **6**a). On the day of heating, a 2 mm thermoseed was inserted into the tumor and heated for 5 min (with a 1 min break after every minute of heating). We observed a dramatic decrease in BLI signal intensity at the tumor site immediately after the heating was completed (Figure 6b,c). In contrast, no change in the tumor BLI signal was recorded in the control group after the thermoseed was implanted (Figure 6b,c). Over the 10 days following treatment, tumor volume of animals in the heated group continuously fell (Figure 6e), and the BLI signal at the tumor site remained at less than 17% of its pretreatment value (Figure 6d). Both measurements rose steadily in the control group, creating clear separations from the heated group (Figure 6d,e). Some heated animals were followed until 33 days after treatment and showed complete ablation of the tumor bulk (Figure 6f). We also conducted histology on tumor samples in which a 1.5 or 2 mm thermoseed had been inserted and heated for 1 min. Representative sections stained with hematoxylin and eosin show clear regions of necrosis (Figure 6g) with approximate diameters of 1.7 and 2.9 mm for the 1.5 and 2 mm thermoseeds, respectively. In the control sample (1.5 mm thermoseed, no AMF), the needle tract through which the thermoseed was inserted is visible, but there is no distinct region of necrosis. Furthermore, when a 2 mm thermoseed was heated for 3 min a larger necrotic region, spanning 4.8 mm, was detected.

## 4 Discussion

This study introduces MINIMA, a novel, minimally invasive, image‐guided technique, whereby an MRI scanner is used to navigate an untethered, ferromagnetic thermoseed through tissue to a target site, before delivering a thermoablative therapy. Using anatomical MR images, an optimum path can be planned from injection site to target, with consideration for intervening tissues and structures. Imaging is then used to monitor the position of the thermoseed along the planned trajectory, enabling full automation of the procedure. This, in addition to a precisely controlled ablation volume, makes MINIMA a promising alternative to surgical or more invasive approaches, minimizing damage to healthy tissue and key functional areas.

Properties of the thermoseed, such as shape, size, and material, can have a significant impact on the imaging, navigation, and heating components. Therefore, careful consideration was taken in the decision to use chrome steel spheres. First, chrome steel has a high saturation magnetization (>1.6 T), which increases the translational forces generated, thus improving the ability to penetrate tissue.^[^ ^18^ ^]^ Second, any nonspherical shape would align its longest axis with the static magnetic field of the MRI system, thus restricting its movement in the transverse plane; a spherical shape enables ease of movement in all directions. An additional factor is thermoseed size. A larger thermoseed will increase both the translational force and heating effect,^[^ ^38^ ^]^ but may increase the size of the artifact in the MR images. This, in combination with the small imaging volume of the preclinical MRI system, limited thermoseed diameter to 3 mm in this study. The imaging volumes of clinical MRI scanners would remove this restriction, allowing larger thermoseeds to be used, potentially improving both the movement and heating capabilities. In terms of clinical relevance, 3 mm thermoseeds are comparable to standard brain biopsy needles and smaller than 5 mm thermoseeds that showed no evidence of gross hemorrhaging when navigated through the canine brain.^[^ ^23^ ^]^

Performing the procedure entirely within an MRI scanner has the inherent advantage of image‐guidance. As observed in our experiments in brain tissue, thermoseed movement can be hindered by certain structures (Figure 3c), highlighting the importance of image‐guided planning of the thermoseed's trajectory, as well as the need for constant location monitoring. Using an MRI localization method,^[^ ^31^ ^]^ our mean error in detecting the position of the thermoseed was no more than 0.27 mm, which is comparable to other validation studies, reporting errors of 0.1–0.3 mm.^[^ ^30^, ^31^ ^]^ This level of accuracy is considered adequate for MINIMA, as the error is smaller than the typical resolution of a clinical 2D MR image (≈1.5 mm), enabling the thermoseed's position to be defined within a single voxel. In addition, the imaging can be performed in less than 1 s,^[^ ^31^, ^39^ ^]^ therefore allowing repeated imaging with no impact on the total procedure duration.

In terms of navigation, we have been able to demonstrate image‐guided movement of a ferromagnetic thermoseed through tissue using the imaging gradients of an MRI system. Our results have shown that increased thermoseed diameter, gradient strength, and duty cycle can all improve the ability to penetrate tissue and the efficiency of movement (Figures 3 and 4 and Figure S1, Supporting Information). However, these larger gradient strengths and duty cycle would require a specialist gradient insert for clinical MRI systems. Existing systems include the Connectom gradient set, which can achieve 300 mT m^−1[^ ^34^ ^]^ and with which we have already demonstrated the ability to move a thermoseed (Figure 4a). In addition, purpose‐built propulsion and imaging gradients that can reach 500 and 200 mT m^−1^, respectively, and operate continuously,^[^ ^40^, ^41^ ^]^ are currently in use. Specifications for targeting gradient coils would be application‐dependent, but these examples give confidence that coils could be designed to deliver efficient movement of ferromagnetic thermoseeds through tissue and provide an ideal starting point for future developments. Should sufficient gradient strengths be achieved, it is also possible to envision MINIMA being applied to other tissue types, such as the prostate or pancreas, which may benefit from a novel minimally invasive cancer therapy. An alternative to moving the thermoseeds through tissue is via the circulatory system, which has previously been shown by navigating a chrome steel sphere in large vessels of a swine^[^ ^15^ ^]^ and using the fringe‐field of an MRI system.^[^ ^22^ ^]^

We have demonstrated that heating thermoseeds by applying an external AMF can induce a controlled region of cell death both in cell culture and in vivo. A variety of thermoseeds, differing in shape, size, and material, have previously been explored for use in magnetic hyperthermia treatment.^[^ ^10^, ^25^, ^29^, ^42^, ^43^, ^44^, ^45^ ^]^ However, these studies have relied on heat transfer mechanisms to ensure ablation of the entire tumor volume, and therefore, have typically used multiple, static thermoseeds positioned throughout the target region and prolonged periods of heating (up to 30 min).^[^ ^27^, ^28^, ^29^ ^]^ With the added benefit of navigation, multiple locations can be ablated using a single thermoseed; this is a similar concept to HIFU, in which the focal lesion is rastered through the tumor to ensure complete coverage. Our cell culture data show that heating a thermoseed for 1 min can achieve 86 mm^3^ of cell death, enabling a 1 mL volume to be ablated within a total of 12 min, which is a clinically relevant procedure time, albeit slower than HIFU.^[^ ^46^, ^47^ ^]^ Our data also show how the ablation volume can be controlled by varying the thermoseed size or heating duration, for example, a 2 mm thermoseed heated for 5 min can ablate up to 3.7 mm away from the thermoseed's surface. This is advantageous should dense tissues be encountered, as the thermoseed may be navigated adjacent to the target tissue for heating. Furthermore, in our in vivo experiments, tumors were completely ablated within 5 min, demonstrating how this method of thermoablation is effective under physiological conditions.

Consideration must be taken when inserting and removing the thermoseed from the patient, due to the fringe field gradients at the entrance of the MRI system. To insert the thermoseed beforehand, it would have to be held in place to prevent unwanted movement during patient entry into the scanner. Alternatively, an MRI compatible stereotactic frame can be used to insert the thermoseed within the bore, similar to those used for MRI‐guided interventions^[^ ^48^, ^49^, ^50^ ^]^ and biopsies.^[^ ^51^ ^]^ A third option is to turn off the magnetic field prior to and following the procedure, allowing the patient to enter and exit the bore with no implication of the fringe field. Although not a common feature of MRI scanners, a clinical scanner can be ramped from zero field to 1.5 T within 45 min (personal communication, Tesla Engineering Ltd.). Although this ramping rate is time‐consuming, magnet design optimization could reduce this time. This has already been achieved preclinically (MR Solutions Ltd.) using a magnet that can ramp from 0 to 3 T within 7.5 min. Using this rampable system, a 3 mm thermoseed did not move during the ramping process (Figure S1, Supporting Information), confirming that this is a safe option for insertion and removal of the thermoseed.

With constant tracking of the thermoseed throughout the procedure, MINIMA has the potential to be implemented with full autonomy. The workflow begins with a prescan, used to plan the most direct path from injection site to tumor, avoiding essential functional structures. The patient will be positioned on the scanner bed and the thermoseed inserted using a biopsy type needle or with introduction through a catheter. In some cases, such as for prostate cancer, it may be possible to inject the thermoseed directly into the tumor. The patient will then enter the MRI system, and an image will be acquired of the surgical volume and registered with the prescan, allowing the thermoseed's position within the tissue to be accurately determined. The thermoseed is then navigated along the planned trajectory using the propulsion gradients. This will be an automated process, consisting of small movements and image acquisition performed in iteration. Once at the target, the iterative process will continue, navigating through the tumor with additional ablation periods. Finally, the thermoseed will be navigated back to the injection site before removal. In a similar fashion, one could envisage using multiple smaller thermoseeds.^[^ ^17^, ^52^ ^]^ Once in the tumor, the thermoseeds would be heated, resulting in thermal ablation of the surrounding tissue.^[^ ^53^, ^54^ ^]^ Following the procedure, the thermoseeds may be accumulated at either the same or a separate site for retrieval.

## 5 Conclusion

In this study, we have demonstrated the MINIMA concept, a minimally invasive, MRI‐driven ablation therapy. By manipulating the magnetic field gradients generated by an MRI system, a ferromagnetic thermoseed can be navigated through brain tissue with submillimeter accuracy. Localized, controllable cell death was also delivered by heating the thermoseed using an external AMF. Given the need to improve oncological effectiveness, this brand‐new, MRI‐based theranostic device, enables a new paradigm in cancer therapy, delivering diagnosis and therapy from a single platform, which may also be applied to other disease areas.

## Conflict of Interest

The authors declare no conflict of interest.

## Supporting information

Supporting Information

## Data Availability

The data that support the findings of this study are available from the corresponding author upon reasonable request.
