# Supplementary material for: Image‐Guided Magnetic Thermoseed Navigation and Tumor Ablation Using a Magnetic Resonance Imaging System
Source: Adv Sci (Weinh). 2022 Feb 2;9(12):2105333. doi: 10.1002/advs.202105333 (PMC9036015; doi:10.1002/advs.202105333)
Supplement: Supplementary file 1 — Supporting Information [file ADVS-9-2105333-s001.pdf]

ADVANCED SCIENCE Open Access

Supporting Information

for *Adv. Sci.*, DOI 10.1002/advs.202105333

Image-Guided Magnetic Thermoseed Navigation and Tumor Ablation Using a Magnetic Resonance Imaging System

*Rebecca R. Baker, Christopher Payne, Yichao Yu, Matin Mohseni, John J. Connell, Fangyu Lin, Ian F. Harrison, Paul Southern, Umesh S. Rudrapatna, Daniel J. Stuckey, Tammy L. Kalber, Bernard Siow, Lewis Thorne, Shonit Punwani, Derek K. Jones, Mark Emberton, Quentin A. Pankhurst and Mark F. Lythgoe**

ADVANCED SCIENCE
Open Access

Supporting Information

for *Adv. Sci.*, DOI: 10.1002/advs.202105333

Image-guided Magnetic Thermoseed Navigation and Tumor Ablation Using an MRI System

*Rebecca R. Baker[#], Christopher Payne[#], Yichao Yu, Matin Mohseni, John J. Connell, Fangyu Lin, Ian F. Harrison, Paul Southern, Umesh S. Rudrapatna, Daniel J. Stuckey, Tammy L. Kalber, Bernard Siow, Lewis Thorne, Shonit Punwani, Derek K. Jones, Mark Emberton, Quentin A. Pankhurst, Mark F. Lythgoe**

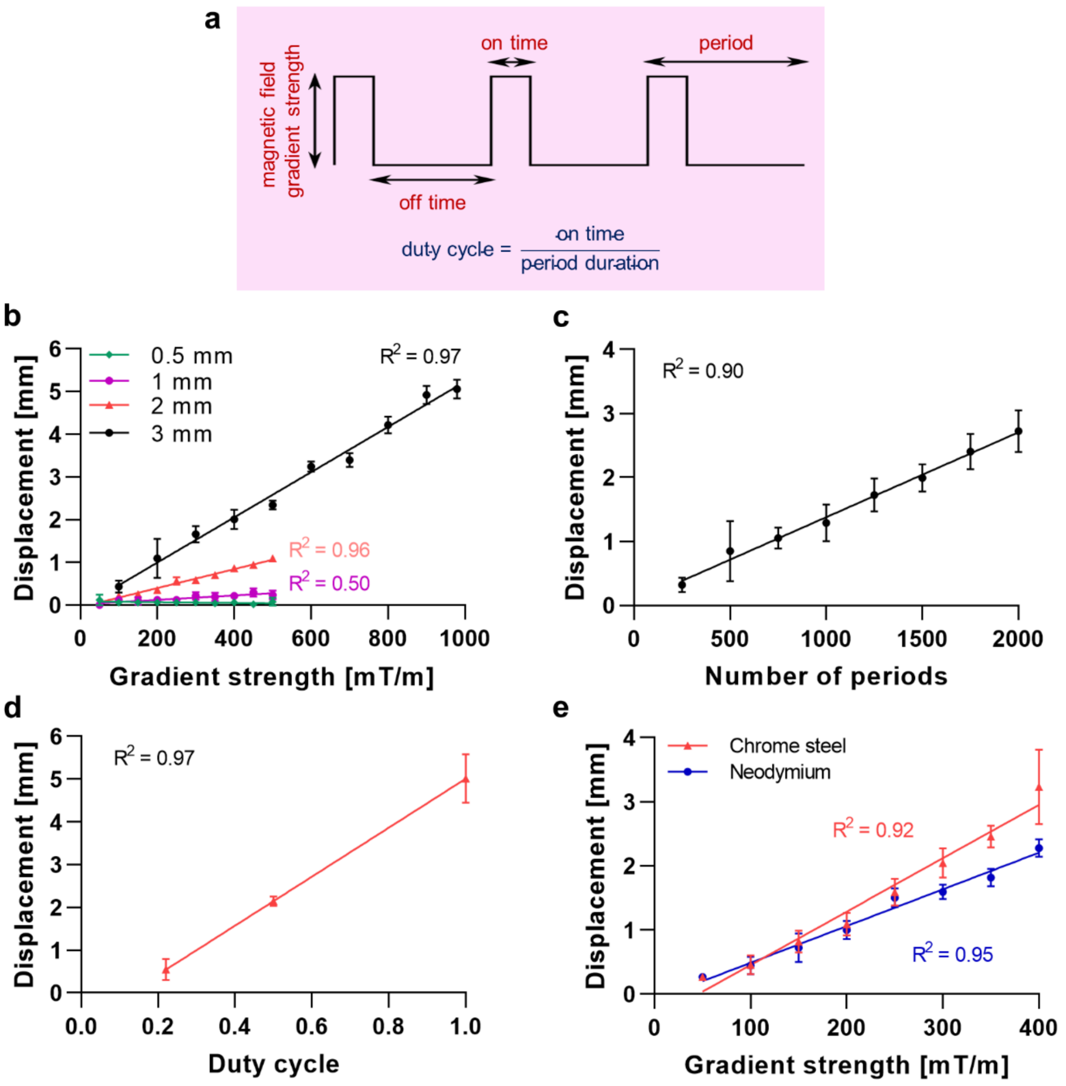

Figure S1. Manipulation of navigation pulse sequence parameters can increase navigation distances. 0.5 – 3 mm diameter thermoseeds were actuated through a viscous medium to assess the effect of different magnetic field gradient parameters on displacement. a) Schematic showing the on-off pattern of magnetic field gradient application, labelled with relevant gradient parameters. Increasing the total time for which the actuation force is applied (total on time) can be achieved by increasing the number of periods or the duty cycle. Displacement is linearly dependent on b) gradient strength (n = 6, $p < 0.0001$ for 1, 2 and 3 mm thermoseeds), c) number of periods (n = 4, $p < 0.0001$) and d) duty cycle (n = 6, $p < 0.0001$). e) Displacement of a 2 mm diameter thermoseed was compared with a Neodymium spherical permanent magnet of the same size, at varying gradient strengths. Linear regression

slopes are significantly different ($n = 6$, $p < 0.0001$). Data analyzed using simple linear regression in all panels.

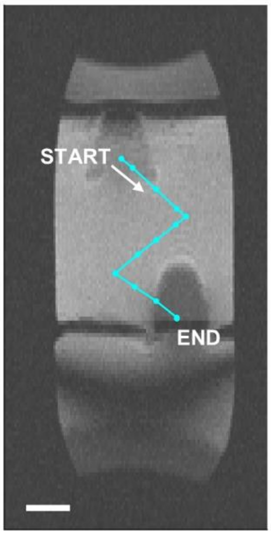

Figure S2. Thermoseeds as small as 0.25 mm can be actuated using a 1 T m^{-1} magnetic field gradient. A 0.25 mm thermoseed was navigated in a zigzag pattern through 0.1 % agar. The path taken by the thermoseed is overlaid onto initial and final position FLASH images. Direction of motion is indicated by the white arrow. Scale bar = 5 mm.

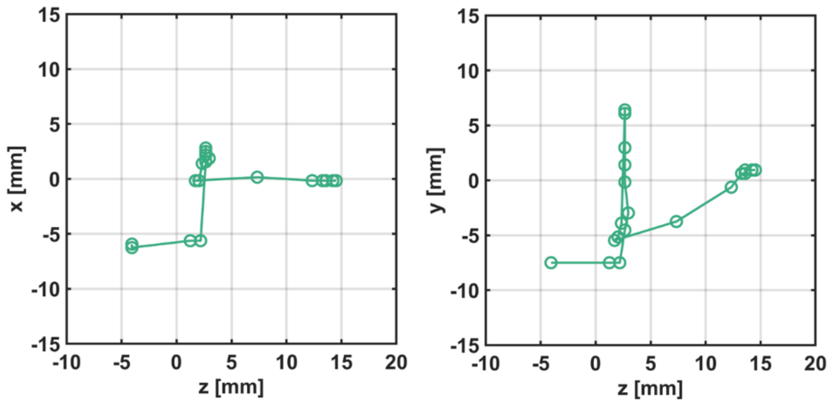

Figure S3. Navigation in 3D. A 1 mm thermoseed was navigated along a 3D path in 0.25 % agar. x and z axes are in the horizontal plane, and y is the vertical axis.

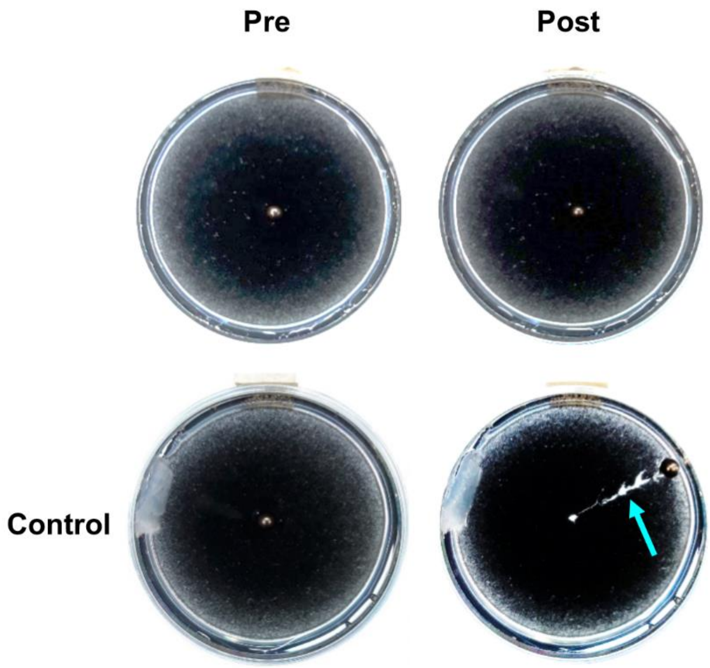

Figure S4. Ramping magnet experiment. A phantom consisting of 0.3% agar mixed with a black pigment was used to observe movement of a 3 mm ferromagnetic thermoseed, whilst ramping an MRI system from zero field up to 3 T and back down. No movement was observed in test samples positioned within the field of view (FOV) of the magnet (top). Control samples (bottom) were positioned outside the FOV, ~13 cm from the isocenter, to demonstrate that movement could be detected using the phantom (arrow).

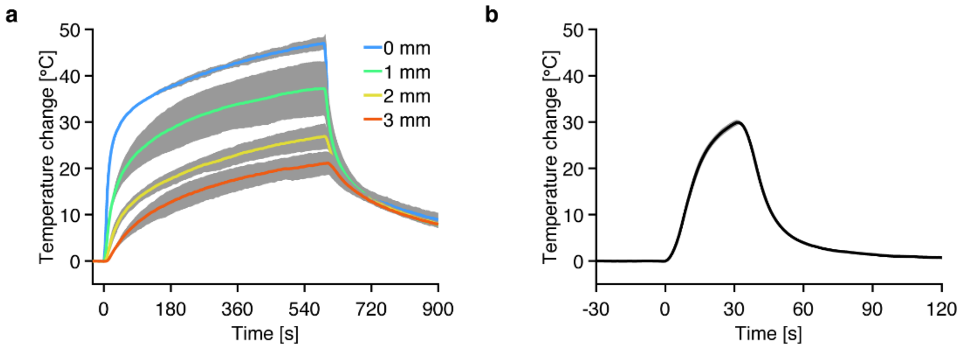

Figure S5. Heating thermoseeds with the MR compatible hyperthermic device. a) Temperature increase when heating in 0.5% agar at different distances from a 2 mm thermoseed. With increasing distance from the thermoseed, the heating rate decreases. Data shown as mean ± S.D., n = 2. b) Change in temperature on the surface of a 2 mm thermoseed that was embedded in 1 % agar and heated in the bore of an MRI scanner, n = 1.

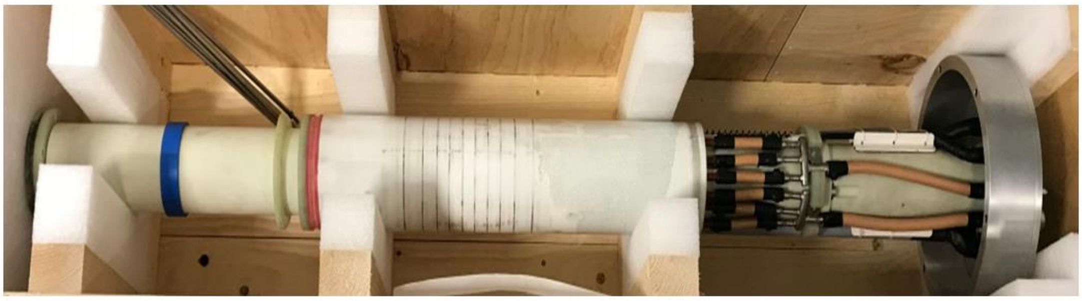

Figure S6. Custom-built MRI propulsion gradient coil. MRI combined propulsion and imaging coil developed in collaboration with Tesla Engineering Ltd. The coil is 786 mm in length, with an internal diameter of 60 mm, designed to fit a 9.4 T Varian Inova MRI scanner. Magnetic field gradients of up to 1150 mT m^{-1} can be achieved along all three axes, when driven by 200A, 350 V amplifiers, with rise times of no more than 21 µs.
